# The Role of Carotid Sinus Nerve Input in the Hypoxic-Hypercapnic Ventilatory Response in Juvenile Rats

**DOI:** 10.3389/fphys.2020.613786

**Published:** 2020-12-17

**Authors:** Paulina M. Getsy, Gregory A. Coffee, Stephen J. Lewis

**Affiliations:** ^1^Department of Pediatrics, Division of Pulmonology, Allergy and Immunology, Case Western Reserve University, Cleveland, OH, United States; ^2^Department of Physiology and Biophysics, Case Western Reserve University, Cleveland, OH, United States; ^3^Department of Pharmacology, Case Western Reserve University, Cleveland, OH, United States

**Keywords:** carotid sinus nerve, hypoxia, hypercapnia, juvenile, rats

## Abstract

In juvenile rats, the carotid body (CB) is the primary sensor of oxygen (O_2_) and a secondary sensor of carbon dioxide (CO_2_) in the blood. The CB communicates to the respiratory pattern generator via the carotid sinus nerve, which terminates within the commissural nucleus tractus solitarius (cNTS). While this is not the only peripheral chemosensory pathway in juvenile rodents, we hypothesize that it has a unique role in determining the interaction between O_2_ and CO_2_, and consequently, the response to hypoxic-hypercapnic gas challenges. The objectives of this study were to determine (1) the ventilatory responses to a poikilocapnic hypoxic (HX) gas challenge, a hypercapnic (HC) gas challenge or a hypoxic-hypercapnic (HH) gas challenge in juvenile rats; and (2) the roles of CSN chemoafferents in the interactions between HX and HC signaling in these rats. Studies were performed on conscious, freely moving juvenile (P25) male Sprague Dawley rats that underwent sham-surgery (SHAM) or bilateral transection of the carotid sinus nerves (CSNX) 4 days previously. Rats were placed in whole-body plethysmographs to record ventilatory parameters (frequency of breathing, tidal volume and minute ventilation). After acclimatization, they were exposed to HX (10% O_2_, 90% N_2_), HC (5% CO_2_, 21% O_2_, 74% N_2_) or HH (5% CO_2_, 10% O_2_, 85% N_2_) gas challenges for 5 min, followed by 15 min of room-air. The major findings were: (1) the HX, HC and HH challenges elicited robust ventilatory responses in SHAM rats; (2) ventilatory responses elicited by HX alone and HC alone were generally additive in SHAM rats; (3) the ventilatory responses to HX, HC and HH were markedly attenuated in CSNX rats compared to SHAM rats; and (4) ventilatory responses elicited by HX alone and HC alone were not additive in CSNX rats. Although the rats responded to HX after CSNX, CB chemoafferent input was necessary for the response to HH challenge. Thus, secondary peripheral chemoreceptors do not compensate for the loss of chemoreceptor input from the CB in juvenile rats.

## Highlights

- Bilateral CSNX blunts, but does not completely block, the hypoxic, hypercapnic and hypoxic-hypercapnic ventilatory responses in juvenile SD rats.
- In SHAM rats, the sum of the responses to hypoxia alone and hypercapnia alone equal the response to hypoxia-hypercapnia.
- In bilateral CSNX rats, the individual ventilatory responses to hypoxia, hypercapnia and hypoxia- hypercapnia were similar.

## Introduction

The detection of oxygen (O_2_), carbon dioxide (CO_2_) and proton (H^+^) levels in arterial blood is crucial for the control of breathing (Prabhakar, 2000; Teppema and Dahan, 2010; Guyenet and Bayliss, 2015; Guyenet et al., 2019). Within seconds, arterial hypoxia (low pO_2_), hypercapnia (high pCO_2_) and/or acidosis (high H^+^ levels) stimulate breathing, which is essential for maintaining proper oxygenation of tissues. This ventilatory adjustment depends critically on the O_2_-sensing ability of the carotid bodies (CBs) and respiratory control brainstem nuclei (Prabhakar, 2000). The CBs are vascularized organs that detect changes in arterial blood pO_2_, pCO_2_ and pH. Decreases in pO_2_ and pH, and/or elevations in pCO_2_ stimulate chemosensitive glomus (type I) cells in the CBs to release neurotransmitters that excite closely apposed nerve terminals of chemoafferent fibers within the carotid sinus nerve (CSN), thereby causing an increase in firing of these afferents (Eyzaguirre and Zapata, 1984; Gonzalez et al., 1994; Peers and Buckler, 1995; Prabhakar, 2000; Tse et al., 2012; Kikuta et al., 2019). CSN chemoafferents then relay these signals to the commissural nucleus tractus solitarius (cNTS) within the brainstem (Song et al., 2011), and increased inputs from these chemoafferent fibers trigger downstream responses to restore arterial blood gas status and hemodynamic homeostasis (Prabhakar, 2000; Nurse, 2005, 2010).

The ventilatory responses to hypoxic (HX) and hypercapnic (HC) gas challenges differ in new-born compared to adult animals. In neonates, the HX ventilatory response appears as a biphasic response comprising of a short initial rise in ventilation (augmentation phase) quickly followed by a secondary roll-off, generally to a level below baseline normoxia (depressant phase) (Bureau et al., 1984; McCooke and Hanson, 1985; Eden and Hanson, 1987; Teppema and Dahan, 2010). Shortly after birth in species such as the pig, cat and rat, the initial rise in minute ventilation (MV) is smaller than the secondary roll-off, resulting in lower baseline ventilation values compared to those under normoxia (Bonora et al., 1984; Eden and Hanson, 1987; Elnazir et al., 1996; Liu et al., 2006; Teppema and Dahan, 2010). Once adulthood is reached, the initial rise in ventilation in response to hypoxia is more pronounced and the secondary depression becomes smaller, resulting in a sustained rise in MV (Easton et al., 1986; Vizek et al., 1987; Maxová and Vízek, 2001; Pokorski and Antosiewicz, 2010; Teppema and Dahan, 2010). The ventilatory response to HC gas challenge during development has been studied extensively in rats and follows a definitive pattern, namely (1) the ventilatory response to HC declines (i.e., shows roll-off) during the challenge in the first post-natal week (Stunden et al., 2001; Putnam et al., 2005), (2) the magnitudes of HC responses are at their lowest points during the second week, and (3) the HC response increases during the third week (i.e., as seen in P21 rats) toward those seen in mature (adult) rats (Stunden et al., 2001; Putnam et al., 2005). The ventilatory response to combined HX and HC (HH) gas challenges (arguably those that induce more physiologically relevant changes in arterial blood-gas chemistry) has not been studied in neonatal rats. However, adult rats exposed to HH gas challenge show enhanced increases in ventilation compared to HX exposures alone, although whether the responses were greater than HC gas challenge alone was not determined (Wakai et al., 2015). Nonetheless, the data strongly suggest that HX and HC ventilatory signaling may be synergistic in rats (Wakai et al., 2015).

The loss of CSN innervation to the CB greatly inhibits compensatory respiratory adjustments in response to HX and HC challenges in neonatal and adult animals. In newborns, the initial rise in ventilation (augmentation phase) in response to acute HX challenge is significantly attenuated after bilateral CSNX (Bureau et al., 1985; Suguihara et al., 1994; Fung et al., 1996; Lowry et al., 1999a, b; Teppema and Dahan, 2010). In adult mammals, bilateral CSNX induces hypoventilation under room-air (Sapru and Krieger, 1977), and diminishes the ventilatory responses to HX and HC challenges (Chalmers et al., 1967; Sapru and Krieger, 1977; Cardenas and Zapata, 1983; Olson et al., 1988; Mouradian et al., 2012; Baby et al., 2018). Within a few weeks after bilateral CSNX there is a noticeable recovery of HX ventilatory responses (Bisgard et al., 1976; Smith and Mills, 1980; Martin-Body et al., 1985, 1986) that are associated with a distinct functional reorganization of central chemoreceptor reflex pathways, including changes in ventilatory pattern and brainstem catecholaminergic activity (Roux et al., 2000). Other reported mechanisms involved in restoration of HX ventilatory response include (1) enhanced aortic body activity (Bisgard et al., 1980; Serra et al., 2002), (2) enhanced chemosensory input from secondary glomus tissue in the head/neck (Martin-Body et al., 1986; Lowry et al., 1999a; Serra et al., 2002), and (3) regeneration of sensory terminals of the carotid sinus nerve, and/or plastic changes in the central respiratory neuronal network involving activation of oxygen-sensing neurons (Smith and Mills, 1980; Lowry et al., 1999b; Serra et al., 2001; Forster, 2003; Mitchell and Johnson, 2003; Teppema and Dahan, 2010).

Along with CB chemoreceptors, mammalian species also possess central chemoreceptors, such as the retrotrapezoid nucleus (RTN), which play a definitive role in breathing modulation, and are key responders to elevated blood pCO_2_ (Nattie, 2006; Guyenet and Bayliss, 2015; Guyenet et al., 2019). The CBs detect arterial pCO_2_ (and pH) and monitor alveolar ventilation, whereas central chemoreceptors detect interstitial fluid pH and monitor the balance of arterial pCO_2_, cerebral blood flow, and cerebral metabolism (Fencl et al., 1966; Dempsey, 2005; Xie et al., 2005; Smith et al., 2006). The time course of responses varies between central and peripheral chemoreceptors, with CBs providing the rapid response to changes in arterial pCO_2_, while central chemoreceptors provide most of the steady-state response (Smith et al., 2006; Gaston et al., 2014). Studies in conscious humans with intact or surgically resected CBs revealed that the fast time constant in response to brief CO_2_ pulses was absent in patients with CB resection, therefore providing strong support for the rapid response function of the CBs (Fatemian et al., 2003).

Although activation of peripheral or central chemoreceptors causes breathing to increase, the type of interaction between the two systems remains unresolved. It has been hypothesized that when a HX and HC stimulus are given together, the interaction between the input from both central and peripheral chemoreceptors can be (1) positive in that one stimulus (e.g., HX or HC) augments the effects of the other in a synergistic/multiplicative, manner or (2) negative in that the inputs are hypo-additive (Eldridge et al., 1981). Studies have shown positive or synergistic interaction between HX and HC at the level of the CB (Eyzaguirre and Lewin, 1961). Other studies have shown that ventilatory responses to a constant CSN stimulation (mimicking HX exposure), decreased progressively as arterial pCO_2_ rose (mimicking a HC exposure), thus suggesting a hypo-additive interaction between the two stimuli (Eldridge, 1974; Kiwull et al., 1976).

Despite evidence that (1) neonatal and adult rats display different HX and HC ventilatory responses, and (2) bilateral CSNX blunts HX and HC ventilatory responses in adult and newborn rats, the effects of bilateral CSNX on HX, HC, or HH gas challenge in juvenile SD rats are unknown. Moreover, the vital question of how HX and HC signaling pathways interact with one another and the role of the CBs in this interaction is not well characterized at any age. To address this, we examined the ventilatory responses elicited by a single 5-min episode of HX, HC or HH gas challenge in conscious, freely moving Sprague Dawley juvenile (postnatal age 25, P25) rats that underwent prior (4 days earlier) sham-operation (SHAM) or bilateral CSNX to disrupt the chemoreceptor reflex pathway. Each gas challenge was given to separate groups of rats (e.g., HX challenge had a group of SHAM and CSNX rats that was separate from the HC and HH challenge) in order to prevent any confounding influences that one gas challenge may have on the other. Our novel findings demonstrating that CSNX blunts the HX-, HC-, and HH-induced ventilatory responses provide the first *in vivo* data showing that hyperventilation in response to these challenges in juvenile rats is dependent on CB chemoafferent signaling. Our results also show that the ventilatory responses to HX alone and HC alone were simply additive in SHAM rats and that this additivity was lost in CSNX rats, suggesting that peripheral CB chemoafferent activity plays an essential role in determining the interactions between peripheral and central chemoafferent pathways controlling ventilation.

## Methods

### Animals and Surgeries

**FIGURE 1:**
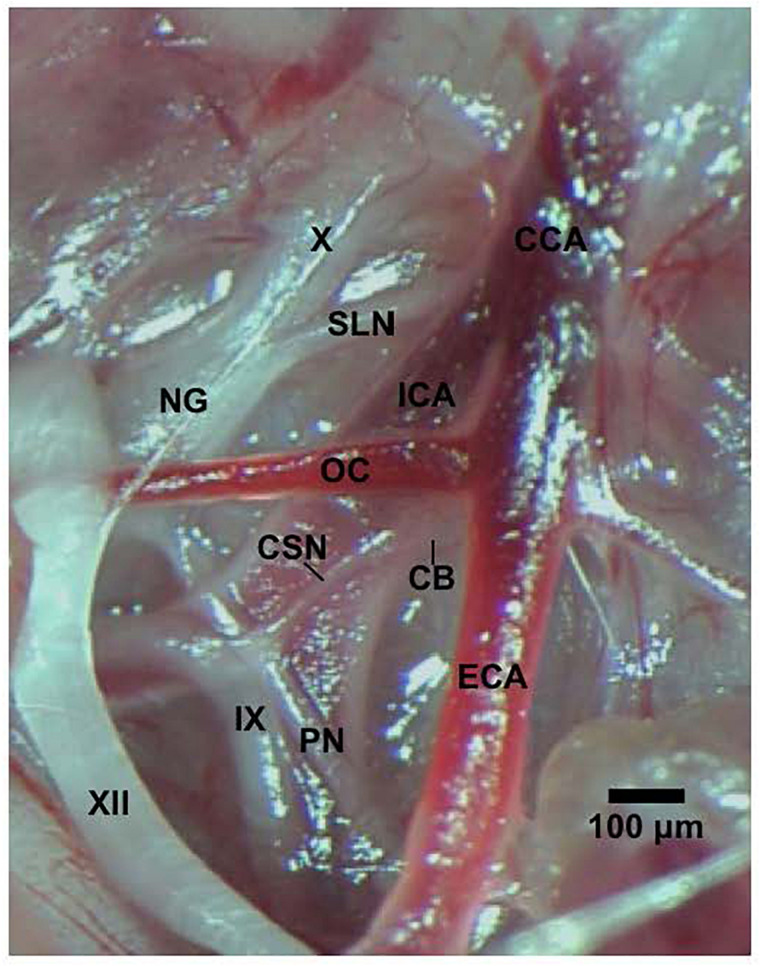
Photograph in a SD male P25 juvenile rat of the carotid sinus nerve (CSN) branching off the glossopharyngeal nerve (IX) and entering the carotid body (CB). The pharyngeal nerve (PN), hypoglossal nerve (XII), nodose ganglion (NG), vagus nerve (X), superior laryngeal nerve (SLN), common carotid artery (CCA), internal (ICA), and external (ECA) carotid arteries, and occipital artery (OC) are also shown. This dissection was done on the left side of the animal. The scale bar is 100 μm.

All studies were carried out in accordance with the National Institute of Health Guide for the Care and Use of Laboratory Animals (NIH Publication No. 80–23) revised in 1996. The protocols were approved by the Institutional Animal Care and Use Committee at Case Western Reserve University (Cleveland, OH, United States). Eighty-two male Sprague Dawley (SD) rats (postnatal age 21, P21) from ENVIGO (Indianapolis, IN, United States) were used in these studies. All of the rats were anesthetized with an intraperitoneal injection of ketamine (80 mg/kg, Ketaset, Zoetis, Parsippany, NJ, United States) and xylazine (10 mg/kg, Akorn Animal Health, Lake Forest, IL, United States), and placed on a surgical station allowing body temperature to be maintained at 37°C via a heating pad (SurgiSuite, Kent Scientific Corporation, Torrington, CT, United States). The surgical plane of anesthesia was checked every 15 min by a toe pinch. Bilateral CSNX and sham-operations (SHAM) were performed. For bilateral CSNX, the left and right CSN was transected at the point where they entered their ipsilateral glossopharyngeal nerve (Figure 1) by procedures detailed previously (Gaston et al., 2014; Baby et al., 2018). For the SHAM procedure, the left and right CSN were identified, but not transected. The rats were allowed 4 days to recover from surgery and were P25 on the day of the experimental study.

### Recording of Ventilatory Parameters

Ventilatory parameters were continuously recorded in freely moving P25 juvenile SHAM or bilateral CSNX rats via whole-body plethysmography technology (Buxco^®^ Small Animal Whole Body Plethysmography, DSI a division of Harvard Biosciences, Inc., St. Paul, MN, United States), as detailed previously (Henderson et al., 2013, 2014; May et al., 2013a, b; Baby et al., 2018). All studies were performed in a quiet laboratory with atmospheric pressure of 760 mmHg (sea-level). The chamber volumes were 0.5 L and the gas flowing through all of the chambers was 0.5 L/min. The chamber temperatures during the acclimatization period were: SHAM and CSNX rats in HX study (26.6 ± 0.1 and 26.7 ± 0.1°C, respectively *P* > 0.05), SHAM and CSNX rats in the HC study (26.2 ± 0.2 and 25.8 ± 0.2°C, respectively *P* > 0.05), and SHAM and CSNX rats in the HH study (26.3 ± 0.2 and 26.3 ± 0.1°C, respectively *P* > 0.05). The chamber humidities during the acclimatization period were: SHAM and CSNX rats in the HX study (62.1 ± 2.4 and 60.8 ± 2.0 %, respectively *P* > 0.05), SHAM and CSNX rats in the HC study (63.2 ± 2.5 and 61.4 ± 2.5 %, respectively *P* > 0.05), and SHAM and CSNX rats in the HH study (62.8 ± 1.3 and 61.1 ± 1.1 %, respectively *P* > 0.05). Directly recorded parameters used in this manuscript were frequency of breathing (fR) and tidal volume (VT). The calculated parameter we used was minute ventilation (MV), which is (fR × VT) (Laferrière et al., 2005; Young et al., 2013). The software constantly corrected digitized values for changes in chamber temperature and humidity, and a rejection algorithm excluded motion-induced artifacts (Henderson et al., 2013, 2014; May et al., 2013a, b; Getsy et al., 2014; Baby et al., 2018).

### Protocols and Data Recording Including Maximal Attainable Responses

The rats were placed in plethysmography chambers to continuously record (breath by breath) ventilatory parameters. The rats were allowed to acclimatize for at least 60 min to allow stable baseline values to be recorded over a 15 min period prior to exposing the rats to HX, HC and HH gas challenges. After the rats where placed in their chamber, they would explore the new environment for about 15-20 min after which time they would usually lay still, periodically grooming themselves and occasionally sniffing. As such, at the time the HX, HC and HH gases were delivered to the chambers, the rats were awake and resting quietly. The behavior of the rats did not change appreciably upon delivery of the HX, HC and HH gas challenges. The occasional rat explored for 15-30 s or groomed for 5-10 s. The rats actually underwent five 5-min episodes of a poikilocapnic HX (10% O_2_, 90% N_2_), HC (5% CO_2_, 21% O_2_, 74% N_2_) or HH (5% CO_2_, 10% O_2_, 85% N_2_) gas challenge each separated by 15 min of room-air. We referred to this as the 5 × 5 HX, HC or HH gas challenge, however, this manuscript will detail the responses that occurred during the first gas challenge only, with findings pertaining to responses that occurred during challenges 2-5 to be detailed in subsequent reports. As such, we designated the responses elicited by the first gas challenge as episode 1 (E1) in this manuscript. The data was continuously recorded before, during the entire 5 × 5 HX, HC or HH gas challenge, and during the 15 min post-challenge periods. For each ventilatory parameter, data points collected during every 15 s epoch were averaged for each rat for graphing and analyses. The maximal values during the HX, HC or HH gas challenge did not necessarily occur during the same 15 s epoch in each rat, and so the maximal values obtained by each rat was also collected. Moreover, the maximal attainable values that occurred throughout the entire study (whether during the 5 × 5 HX, HC or HH gas challenge or subsequent RA phases) were determined. Every 15 s value was sorted from largest to smallest and the top ten highest values were averaged to provide the maximal attainable fR, VT, and MV values.

### Data Analysis

All values are expressed as mean ± SEM. The data were analyzed by one-way or two-way analysis of variance tests that were followed by Student’s modified *t*-tests with Bonferroni corrections for multiple comparisons between means using the error mean square terms arising from the ANOVA tests (Wallenstein et al., 1980) as detailed previously (May et al., 2013a, b). The ANOVAs and the multiple comparisons tests were performed using the statistical programs, JMP Statistical Analysis Software (JMP, Cary, NC, United States) and Sigma XL (SigmaStat, Canada, Kitchener, ON, Canada). In every instance, these programs tested for normality and homogeneity of variances before running the ANOVAs. All data sets were normally distributed with homogenous variances and therefore suitable for ANOVA testing. For each rat, the Pre-values for each parameter were determined as the average of the values recorded over the 90 s (i.e., six 15-s epochs) immediately prior to each gas challenge. These resting (baseline) data, and the data recorded over the entire acclimatization period, from the six groups of rats (SHAM and CSNX groups for the HX, HC and HH gas challenges) were analyzed by one-way ANOVA with Bonferroni corrections. The arithmetic changes in ventilatory parameters elicited by the gas challenges at 15, 30, 45, and 60 s were determined by simply subtracting the actual values obtained at these time points for each rat from the Pre-value recorded for each rat. Group data for SHAM or CSNX groups (12 values, 4 times × 3 gas challenges per group) were analyzed by two-way ANOVA with Bonferroni corrections. These individual values were summed to provide the total of these responses. The resulting three values for each SHAM and CSNX groups (HX, HC and HH values) were then analyzed by one-way ANOVA with Bonferroni corrections. The total arithmetic changes in ventilatory parameters in SHAM and CSNX rats were calculated from Pre-values and summed to provide the total response (i.e., sum of 20 individual 15 s epochs for the 5 min challenge). The resulting three values for each of the SHAM and CSNX groups (HX, HC and HH values) were then analyzed by one-way ANOVA with Bonferroni corrections. To compare the ventilatory parameters recorded during the actual HH challenge with the additive HX+HC values, we performed simple addition of responses obtained from the rats that received the HX challenge and from rats that received the HC challenge. These data are presented as mean of summed values ± SEM (10% of mean). The 10% SEM value was chosen because it is consistent with the SEM values of the actual data. It is important to note that none of the theoretical HX+HC values were used in any of the statistical analyses as the HX+HC values were only shown to support the interpretation of the actual HX, HC and HH responses. For descriptive purpose only, we chose a percentage difference of greater or less than 25% of the actual HH values to be a noticeable change from the HX+HC values and therefore subject to discussion.

## Results

### Resting Parameters

A summary of the descriptors and baseline ventilatory parameters for SHAM and CSNX rats is provided in Table 1. The ages and body weights of the SHAM and CSNX groups for the HX, HC and HH gas challenges were statistically similar to one another (upper rows of Table 1). As such, no corrections for differences in body weights were applied to the ventilatory data. The ventilatory parameters shown in the middle rows of Table 1 are those recorded throughout the 15-min baseline period, and show that fR, VT and MV, in each CSNX group for the HX (poikilocapnic), HC and HH gas challenge were significantly smaller compared to those in the respective SHAM group (*P* < 0.05, CSNX *versus* SHAM in HX, HC and HH for all comparisons). The ventilatory values in the bottom rows of Table 1 represent those recorded immediately (2-3 min) before each HX, HC and HH gas challenge in SHAM and CSNX groups (i.e., Pre-values). Pre-values were significantly depressed for fR, VT, and MV in each CSNX group (i.e., each CSNX group for HX, HC, and HH gas challenge) compared to the respective SHAM group.

**TABLE 1:** Baseline parameters in sham-operated rats and in those with bilateral transection of the carotid sinus nerve.

Parameter	Group	HX Challenge	HC Challenge	HH Challenge
Number of rats	SHAM	14	12	15
	CSNX	14	12	15
Age, days post birth	SHAM	25	25	25
	CSNX	25	25	25
Body Weight, grams	SHAM	60 ± 3	68 ± 3	61 ± 2
	CSNX	62 ± 4	65 ± 2	59 ± 2
**Values recorded throughout the acclimatization period**				
Frequency, breaths/min	SHAM	123 ± 5	120 ± 2	125 ± 3
	CSNX	109 ± 3*	114 ± 2*	112 ± 3*
Tidal Volume, ml	SHAM	0.63 ± 0.02	0.68 ± 0.02	0.62 ± 0.01
	CSNX	0.55 ± 0.01*	0.59 ± 0.01*	0.56 ± 0.01*
Minute Ventilation, ml/min	SHAM	72 ± 4	79 ± 2	75 ± 1
	CSNX	59 ± 2*	65 ± 2*	60 ± 2*
**Values recorded over the 90 s prior to the gas challenges (i.e., Pre-values)**				
Frequency, breaths/min	SHAM	113 ± 4	117 ± 2	119 ± 3
	CSNX	100 ± 2*	106 ± 2*	106 ± 3*
Tidal Volume, ml	SHAM	0.60 ± 0.02	0.66 ± 0.01	0.60 ± 0.01
	CSNX	0.55 ± 0.01*	0.58 ± 0.01*	0.55 ± 0.01*
Minute Ventilation, ml/min	SHAM	69 ± 3	79 ± 1	73 ± 2
	CSNX	56 ± 2*	63 ± 1*	59 ± 2*

SHAM, sham-operated rats. CSNX, rats with bilateral carotid sinus nerve transection. HX, hypoxic; HC, hypercapnic; HH, hypoxic-hypercapnic. Data are presented as mean ± SEM. * P < 0.05, CSNX versus SHAM.

### Example Traces Recorded at Baseline and During HX, HC, or HH Gas Challenges

**FIGURE 2:**
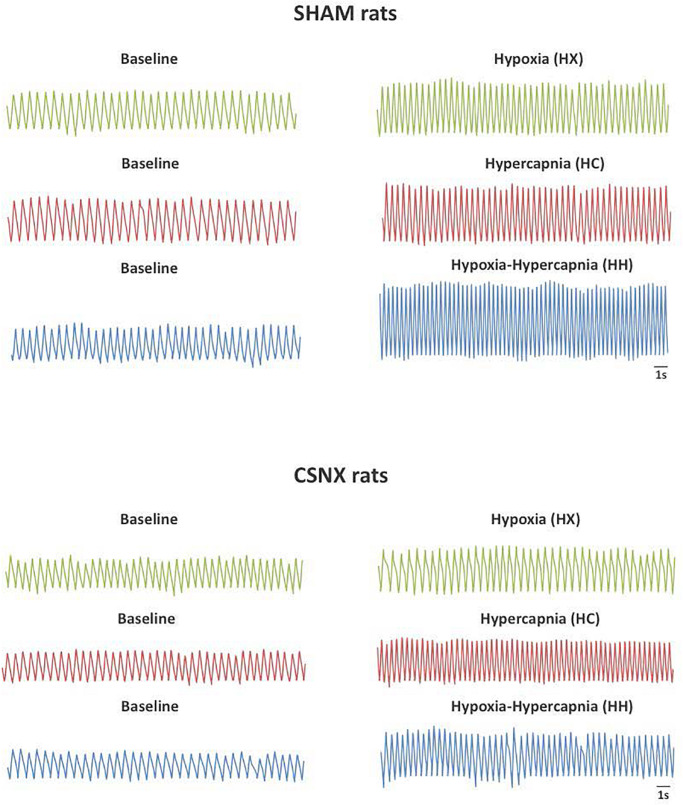
Representative plethysmography traces from sham-operated (SHAM) P25 rats (**Top panel**) and bilateral carotid sinus nerve transection (CSNX) (**Bottom panel**) P25 rats recorded at baseline and hypoxic (HX; 10% O_2_, 90% N_2_), hypercapnic (HC; 5% CO_2_, 21% O_2_, 74% N_2_), or hypoxic-hypercapnic (HH; 5% CO_2_, 10% O_2_, 85% N_2_) gas challenges.

Representative sections of plethysmography traces from SHAM and CSNX rats are shown in Figure 2. In response to HX, the fR in the SHAM rat increased from 102 to 144 breaths/min (+42 breaths/min). The increase in fR in the CSNX rat was smaller (from 102 to 108 breaths/min, +6 breaths/min). In response to HC, fR in the SHAM rat increased from 96 to 144 breaths/min (+48 breaths/min). The increase in fR in the CSNX rat was similar (from 108 to 162 breaths/min, +54 breaths/min). In response to HH, fR in the SHAM rat increased from 102 to 186 breaths/min (+84 breaths/min). The increase in fR in the CSNX rat was smaller (from 102 to 138 breaths/min, +36 breaths/min). The increase in fR to HH in the SHAM rat (+84 breaths/min) was similar to simple addition of the HX and HC responses (42 + 48 = 90 breaths/min), whereas the HH response in the CSNX rat (+36 breaths/min) was much smaller than the simple addition of the HX and HC responses (6 + 54 = 60 breaths/min).

### Comparison of the HX-, HC-, and HH-Induced Responses to the Maximal Attainable Values

The detailed changes in ventilatory responses to HX, HC and HH gas challenges will be described below. It is important to note that these challenges, including HH, did not produce changes in ventilatory parameters that approached the maximal attainable responses recorded in these rats throughout the entire study. Specifically, the Pre-values, the maximal values recorded during the entire study (maximum), and the peak values obtained during the HX, HC or HH are shown in Table 2. For fR (top rows), VT (middle rows), and MV (bottom rows), the delta values for the gas challenge responses were all smaller than the delta values for the maximal attainable responses in the CSNX group compared to the SHAM (*P* < 0.05, for all comparisons).

**TABLE 2:** Maximal attainable values and maximal values recorded during the gas challenges.

Parameter	Gas	Group	Pre-value	Maximum	Gas challenge	Maximum delta	Gas challenge delta
fR (bpm)	HX	SHAM	113 ± 4	355 ± 16	193 ± 10	+ 242 ± 16^†^	+ 80 ± 9^†,‡^
		CSNX	100 ± 2*	387 ± 9	164 ± 8	+ 287 ± 10^†^	+ 64 ± 8^†,‡^
	HC	SHAM	117 ± 2	351 ± 15	222 ± 18	+ 234 ± 17^†^	+ 105 ± 18^†,‡^
		CSNX	106 ± 2*	363 ± 15	190 ± 13	+ 257 ± 16^†^	+ 84 ± 14^†,‡^
	HH	SHAM	119 ± 3	343 ± 19	246 ± 11	+ 224 ± 19^†^	+ 126 ± 10^†,‡^
		CSNX	106 ± 3*	377 ± 14	211 ± 16	+ 271 ± 14^†^	+ 105 ± 15^†,‡^
VT (ml)	HX	SHAM	0.60 ± 0.02	1.12 ± 0.05	0.97 ± 0.04	+ 0.53 ± 0.04^†^	+ 0.37 ± 0.03^†,‡^
		CSNX	0.55 ± 0.01*	1.09 ± 0.05	0.82 ± 0.03	+ 0.54 ± 0.05^†^	+ 0.27 ± 0.03^†,‡^
	HC	SHAM	0.66 ± 0.01	1.14 ± 0.04	1.03 ± 0.04	+ 0.48 ± 0.03^†^	+ 0.37 ± 0.03^†,‡^
		CSNX	0.58 ± 0.01*	1.00 ± 0.02	0.88 ± 0.03	+ 0.42 ± 0.02^†^	+ 0.30 ± 0.02^†,‡^
	HH	SHAM	0.60 ± 0.01	1.35 ± 0.04	1.22 ± 0.04	+ 0.74 ± 0.04^†^	+ 0.62 ± 0.03^†,‡^
		CSNX	0.55 ± 0.01*	1.00 ± 0.03	0.87 ± 0.02	0.45 ± 0.03^†^	+ 0.32 ± 0.02^†,‡^
MV (ml/min)	HX	SHAM	69 ± 3	222 ± 13	154 ± 6	+ 153 ± 11^†^	+ 84 ± 5^†,‡^
		CSNX	56 ± 2*	234 ± 13	103 ± 5	+ 178 ± 12^†^	+ 46 ± 5^†,‡^
	HC	SHAM	79 ± 1	238 ± 12	178 ± 14	+ 159 ± 11^†^	+ 99 ± 13^†,‡^
		CSNX	63 ± 1*	203 ± 13	133 ± 6	+ 140 ± 13^†^	+ 70 ± 5^†,‡^
	HH	SHAM	73 ± 1	262 ± 5	240 ± 4	+ 190 ± 6^†^	+ 168 ± 4^†,‡^
		CSNX	59 ± 2*	198 ± 6	145 ± 7	+ 138 ± 6^†^	+ 85 ± 6^†,‡^

SHAM, sham-operated rats. CSNX, rats with bilateral carotid sinus nerve transection. fR (bpm), frequency of breathing (breaths/min); VT, tidal volume; MV, minute ventilation. HX, hypoxic gas challenge; HC, hypercapnic gas challenge; HH, hypoxic-hypercapnic gas challenge. Data are presented as mean ± SEM. *P < 0.05, Pre-values, CSNX versus SHAM. ^†^P < 0.05, delta responses, significant changes from Pre-values. ^‡^P < 0.05, Hypoxia delta response versus maximal attainable delta response.

### Frequency of Breathing (fR) in SHAM and CSNX Rats During HX, HC or HH Gas Challenges

**FIGURE 3:**
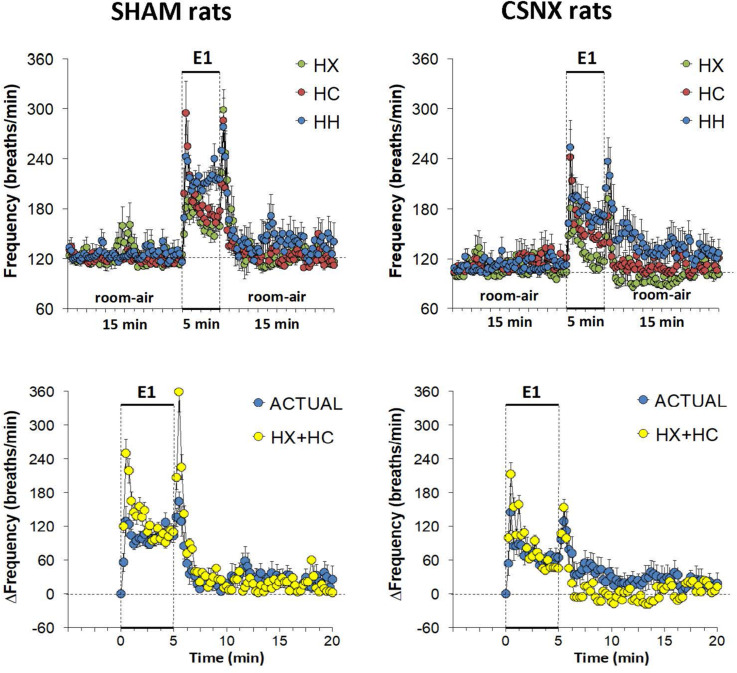
**Top panels:** Frequency of breathing (fR) values averaged every 15 s in freely moving sham-operated (SHAM) rats and in rats with bilateral carotid sinus nerve transection (CSNX) during exposure to a single episode (E1) of hypoxic (HX; 10% O_2_, 90% N_2_), hypercapnic (HC; 5% CO_2_, 21% O_2_, 74% N_2_), or hypoxic-hypercapnic (HH; 5% CO_2_, 10% O_2_, 85% N_2_) gas challenge of 5 min in duration, followed by 15 min of room-air. The data are presented as mean ± SEM. **Bottom panels:** Changes in fR in SHAM and CSNX rats during the actual HH gas challenge compared to addition of HX+HC values. For the actual HH values, data are presented as mean ± SEM and for the HX+HC values, data are presented as the mean ± SEM (10% of mean).

The top panels of Figure 3 summarize fR values of SHAM and CSNX rats before, during, and after exposure to a 5 min HX, HC or HH gas challenge. In SHAM rats (upper left panel), HX and HC elicited robust increases in fR that appeared to display roll-off toward the end of the challenge. HH in SHAM rats elicited a robust increase in fR that did not display roll-off. In CSNX rats (upper right panel), HX elicited an increase in fR that was subject to a more pronounced roll-off than in SHAM rats. The increase in fR elicited by HC in CSNX rats looked comparable to that in SHAM rats, whereas the increase in fR elicited by HH challenge looked substantially smaller in CSNX rats than SHAM rats. The bottom panels of Figure 3 summarize the arithmetic changes in fR that occurred throughout the actual HH protocol, and simple addition of HX and HC values (HX+HC, SEM values presented as 10% of the mean to provide perspective). The actual HH values in SHAM rats (bottom left panel) were smaller than the HX+HC values during the initial phases of the HH gas challenge compared to HX+HC, and the initial seconds upon return to room-air. In SHAM rats, the maximal attainable changes in fR during the entire HH protocol was +224 ± 19 breaths/min (Table 2), which was much higher than the changes in the HH gas challenge (+126 ± 10 breaths/min) (Table 2). In SHAM rats, the summed changes in HX and HC responses (+80 ± 9 and +105 ± 18 breaths/min, respectively; +185 breaths/min upon addition) was smaller than the maximal attainable changes in fR during the entire HH protocol (+224 ± 19 breaths/min). In contrast to SHAM rats, the HH and HX+HC responses in CSNX rats (bottom right panel) were similar to one another. For CSNX rats, the maximal attainable changes in fR during the entire HH protocol (+271 ± 14 breaths/min) (Table 2), was again substantially higher than those observed during the HH gas challenge (+105 ± 15 breaths/min) or those obtained by summation of the HX and HC responses (+64 ± 9 and +84 ± 14 breaths/min, respectively; +148 breaths/min upon addition).

**FIGURE 4:**
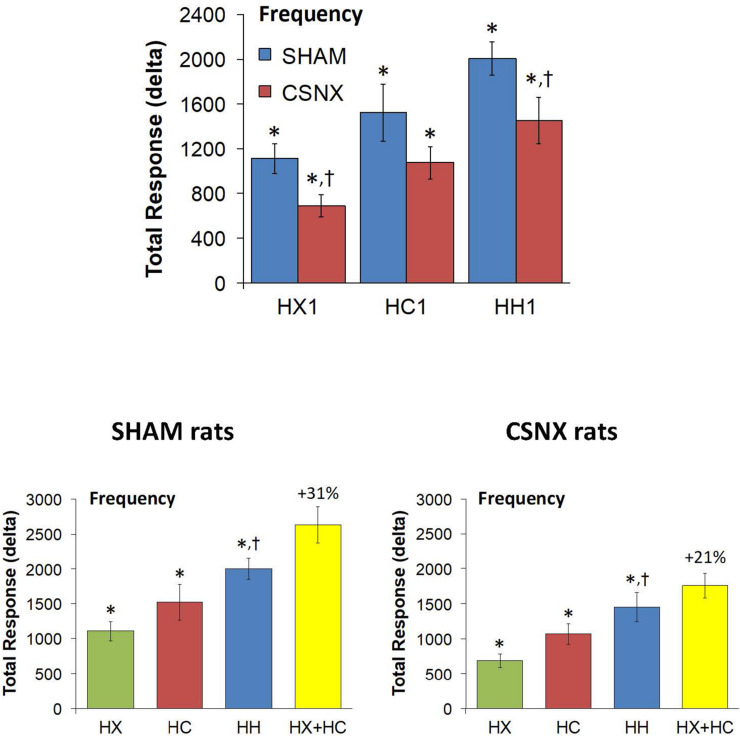
**Top panels:** Total arithmetic changes in frequency of breathing (fR) that occurred during exposure to a single hypoxic (HX1, 10% O_2_, 90% N_2_), hypercapnic (HC1, 5% CO_2_, 21% O_2_, 74% N_2_) or hypoxic-hypercapnic (HH1, 5% CO_2_, 10% O_2_, 85% N_2_) gas challenge in SHAM and CSNX rats. The data are presented as mean ± SEM. ^∗^*P* < 0.05, significant response from Pre-value. ^†^*P* < 0.05, CSNX *versus* SHAM. **Bottom panels:** Total arithmetic changes in fR values in SHAM (left panel) and CSNX (right panel) rats during exposure to the HX, HC or HH gas challenges. The sum of HX and HC values (HX+HC) expressed as mean ± SEM (10% of mean) are shown. The figures in parentheses above the HX+HC columns are the percentage (%) differences between actual HH values and HX+HC values. ^∗^*P* < 0.05, significant response from Pre-value. ^†^*P* < 0.05, HH *versus* HX.

The top panels of Figure 4 compare the total increases in fR that occurred during the single HX, HC or HH gas challenge in SHAM and CSNX rats. All three challenges elicited substantial increases in fR from Pre-values. The increase in fR elicited by HX was smaller in CSNX rats than in SHAM rats. The increase in fR during HC were not statistically smaller in CSNX rats compared to SHAM rats. The increase in fR during HH in CSNX rats was smaller than in SHAM rats. Table 3 shows the arithmetic differences between the total 5 min HX, HC and HH fR responses in SHAM and CSNX rats, with the (-) negative values meaning that the responses in CSNX rats were smaller than in SHAM rats. The differences in responses of CSNX and SHAM rats for the additive HX+HC values are also shown. The actual fR differences in HH responses between SHAM and CSNX rats were far smaller than predicted by the addition of HX and HC (HX+HC) responses. The bottom panels of Figure 4 summarize the total fR responses elicited by HX, HC and HH in SHAM rats (bottom left panel) and CSNX rats (bottom right panel). The HX+HC values are also shown, with the values above the columns noting the percentage difference between the actual HH values and HX+HC values. In SHAM rats, the fR response elicited by the actual HH gas challenge was smaller than the summed HX+HC values. In the CSNX rats, the fR response elicited by the actual HH gas challenge was similar to the summed HX+HC values, suggesting that fR is less than additive in SHAM rats and mostly additive in CSNX rats.

**TABLE 3:** Arithmetic differences between the responses observed in sham-operated rats and those with bilateral transection of the carotid sinus nerve.

	Frequency (breaths/min)		Tidal volume (ml)		Minute ventilation (ml/min)	
	Delta ACTUAL	Delta HX+HC	Delta ACTUAL	Delta HX+HC	Delta ACTUAL	Delta HX+HC
HX 1	−419		−2.01		−667	
HC 1	−451		−0.43		−635	
HH 1	−554	−870	−4.65	−2.44	−1448	−1302

*The data are presented as the mean of the differences in frequency of breathing (breaths/min), tidal volume (ml) and minute ventilation (ml/min) between the bilateral carotid sinus nerve transected rats and the sham-operated rats. HX, hypoxic gas challenge; HC, hypercapnic gas challenge; HH, hypoxic-hypercapnic gas challenge.*

**FIGURE 5:**
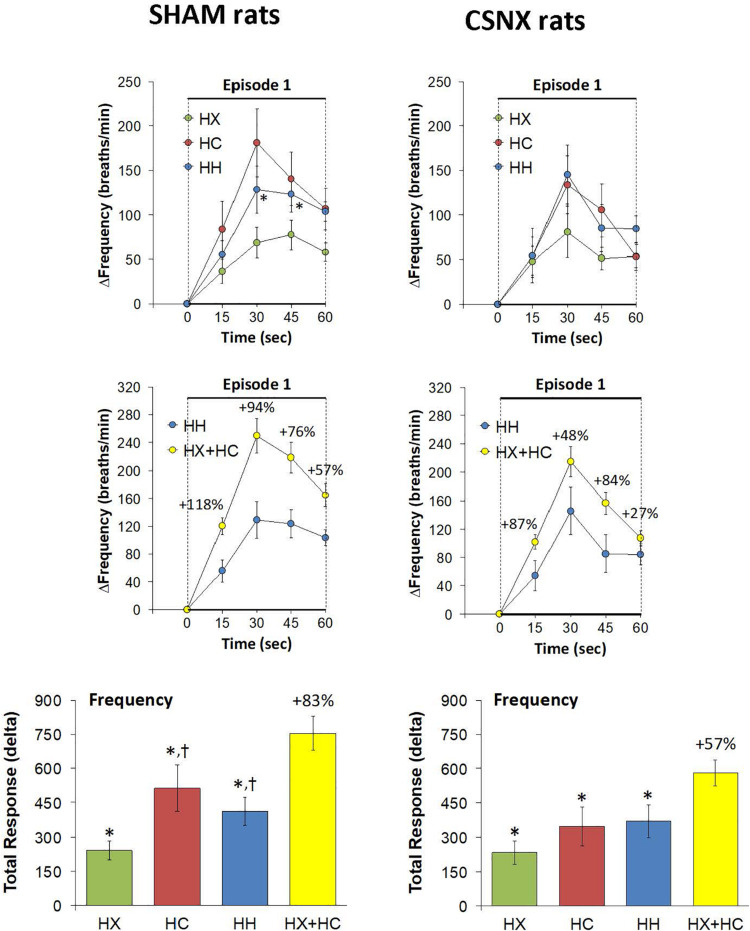
**Top panels:** Arithmetic changes in frequency of breathing (fR) in freely moving sham-operated (SHAM) rats and in rats with bilateral carotid sinus nerve transection (CSNX) during the first 60 s of exposure to a single hypoxic (HX, 10% O_2_, 90% N_2_), hypercapnic (HC, 5% CO_2_, 21% O_2_, 74% N_2_), or hypoxic-hypercapnic (HH, 5% CO_2_, 10% O_2_, 85% N_2_) gas challenge (Episode 1 (E1)). Data are presented as mean ± SEM. ^∗^*P* < 0.05, HH versus HX. ^†^*P* < 0.05, HH *versus* HC. **Middle panels:** Arithmetic changes in fR in SHAM and CSNX rats during the first 60 s of the actual hypoxic-hypercapnic (HH) gas challenge values compared to the addition of the HX and HC (HX+HC) gas challenge values. For the actual HH values, data are shown as mean ± SEM and for HX+HC values, data are presented as the mean ± SEM (10% of mean). **Bottom panels:** Total arithmetic changes in fR in SHAM and CSNX rats during the first 60 s of exposure to HX, HC or HH gas challenges. The data are presented as mean ± SEM. The sum of the HX and HC values (HX+HC), expressed as mean ± SEM (10% of mean), are also shown. The numbers above the HX+HC columns are the percentage (%) differences between the HX+HC values and actual HH values. ^∗^*P* < 0.05, significant response from Pre-value. ^†^*P* < 0.05, HH *versus* HX.

Rapid responses to HX, HC and HH would be expected to involve CB chemoafferents. As such, we focused on the fR responses that occurred during the first 60 s of the HX, HC and HH gas challenge in SHAM and CSNX rats. The changes in fR that occurred during the first 60 s of the HX, HC and HH gas challenges are shown in the top panels of Figure 5. For SHAM rats (top left panel), the initial increases in fR elicited by HX were less than those elicited by the HC or HH, whereas there were minimal differences between HX, HC and HH responses in CSNX rats (top right panel). Overall, the responses during the first 60 s of HX, HC and HH were similar in SHAM and CSNX rats. Next, we wanted to determine whether the changes in magnitude of the actual fR response during the first 60 s of the HH gas challenge was simply additive of those elicited by the HX and HC alone. As seen in the middle panels of Figure 5, the actual HH values in SHAM rats (middle left panel) were substantially less than those of the summed HX+HC values. The actual HH values in CSNX rats (middle right panel) were somewhat less than those of the added HX+HC values. The bottom panels of Figure 5 show the total fR responses that occurred during the first 60 s of HX, HC, and HH gas challenges in SHAM and CSNX rats. Again, for SHAM rats (bottom left panel), the increase in fR during the first 60 s elicited by HH was less than would be expected by simple addition of the HX and HC responses. This was also true for CSNX rats (bottom right panel). The clear impression given by the data is that there is a negative interaction between HX and HC signaling pathways during the first 60 s of a HH gas challenge, and this interaction is independent of the presence or absence of the CB-CSN complex.

### Tidal Volume (VT) in SHAM and CSNX Rats During HX, HC or HH Gas Challenges

**FIGURE 6:**
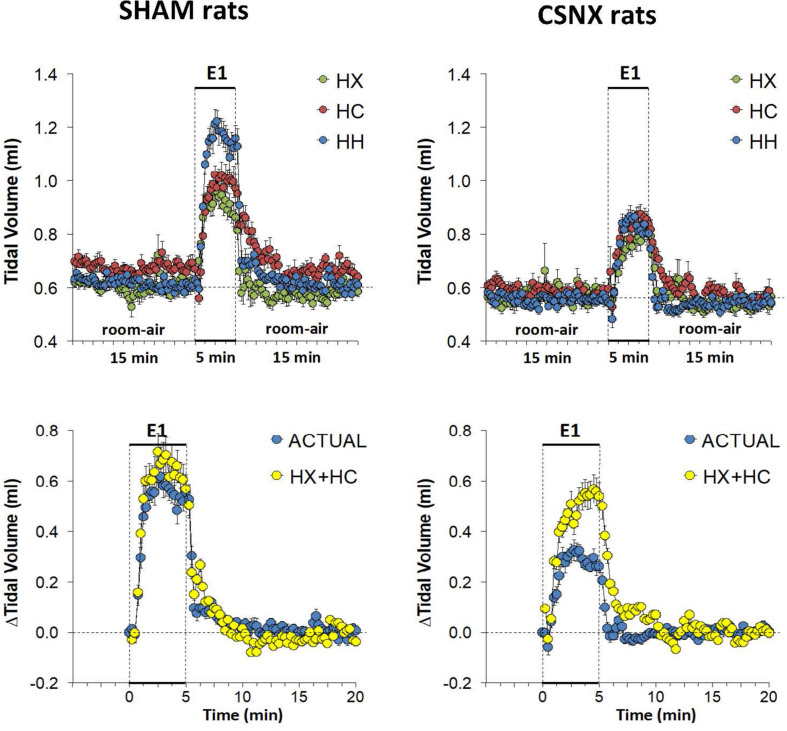
**Top panels:** Tidal volume (VT) values averaged every 15 s in freely moving sham-operated (SHAM) rats and in rats with bilateral carotid sinus nerve transection (CSNX) during exposure to a single episode (E1) of hypoxic (HX; 10% O_2_, 90% N_2_), hypercapnic (HC; 5% CO_2_, 21% O_2_, 74% N_2_), or hypoxic-hypercapnic (HH; 5% CO_2_, 10% O_2_, 85% N_2_) gas challenge of 5 min in duration, followed by 15 min of room-air. The data are presented as mean ± SEM. **Bottom panels:** Changes in VT in SHAM and CSNX rats during the actual HH gas challenge compared to addition of HX+HC values. For the actual HH values, data are presented as mean ± SEM and for the HX+HC values, data are presented as the mean ± SEM (10% of mean).

The top panels of Figure 6 summarize the VT values recorded in SHAM and CSNX rats before, during, and after exposure to 5 min HX, HC or HH gas challenge. In SHAM rats (top left panel), HX and HC gas challenges elicited robust and sustained increases in VT that were similar to one another. HH gas challenge also elicited a sustained increase in VT that appeared greater than those elicited by HX or HC. In contrast, the increases in VT elicited by HX, HC and HH gas challenges in CSNX rats (top right panel) were similar to one another, and all smaller than those in SHAM rats. The bottom panels of Figure 6 summarize the arithmetic changes in VT that occurred throughout the actual HH gas challenge and the simple addition of HX and HC values (HX+HC). In SHAM rats (bottom left panel), the actual HH values and additive HX+HC values were remarkably similar to one another. The maximal attainable changes in VT in the SHAM rat during the entire HH protocol, was +0.74 ± 0.04 ml (Table 2), which was higher than the changes during the HH gas challenge (+0.62 ± 0.03 ml), but equal to the sum of the HX and HC (HX+HC) responses (+0.37 ± 0.03 ml and +0.37 ± 0.03 ml, respectively; +0.74 ml upon addition). In contrast, CSNX rats (bottom right panel), the actual HH VT values were substantially smaller than the additive HX+HC values. The maximal attainable changes in VT in CSNX rats during the entire HH protocol, was +0.45 ± 0.03 ml (Table 2), which was higher than the changes during the HH gas challenge (+0.32 ± 0.02 ml), and lower than the sum of HX and HC (HX+HC) responses (+0.27 ± 0.03 ml and +0.30 ± 0.02 ml, respectively; +0.57 ml upon addition).

**FIGURE 7:**
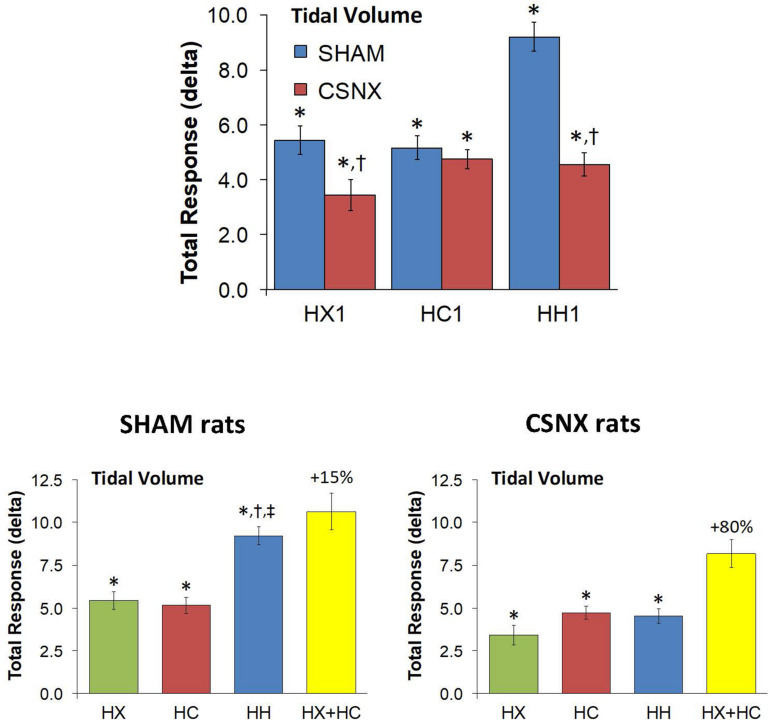
**Top panels:** Total arithmetic changes in tidal volume (VT) that occurred during exposure to a single hypoxic (HX1, 10% O_2_, 90% N_2_), hypercapnic (HC1, 5% CO_2_, 21% O_2_, 74% N_2_) or hypoxic-hypercapnic (HH1, 5% CO_2_, 10% O_2_, 85% N_2_) gas challenge in SHAM and CSNX rats. The data are presented as mean ± SEM. ^∗^*P* < 0.05, significant response from Pre-value. ^†^*P* < 0.05, CSNX *versus* SHAM. **Bottom panels:** Total arithmetic changes in VT values in SHAM (left panel) and CSNX (right panel) rats during exposure to HX, HC or HH gas challenges. The sum of the HX and HC values (HX+HC) expressed as mean ± SEM (10% of mean) are shown. The figures in parentheses above the HX+HC columns are the percentage (%) differences between actual HH values and HX+HC values. ^∗^*P* < 0.05, significant response from Pre-value. ^†^*P* < 0.05, HH *versus* HX. ^‡^*P* < 0.05, HH *versus* HC.

The top panel of Figure 7 compares the total increases in VT that occurred during the single HX, HC or HH gas challenge in SHAM and CSNX rats. The total increases in VT elicited by HX were significantly smaller in CSNX rats than SHAM rats, whereas the VT responses during HC were similar in both groups. The total increases in VT elicited by HH were markedly smaller in CSNX rats than in SHAM rats. As shown in Table 3, the arithmetic differences in VT between SHAM and CSNX rats for HX of -2.01 ml and for HC of -0.43 ml would add to -2.44 ml, whereas the difference for the actual HH was -4.65 ml, suggesting that the actual VT difference in HH responses between SHAM and CSNX rats were far greater than predicted by addition of HX and HC (HX+HC) responses. The bottom panels of Figure 7 summarize the total VT responses elicited HX, HC and HH in SHAM rats (bottom left panel) and CSNX rats (bottom right panel). The HX+HC values are also shown with figures above the HX+HC columns showing the percentage difference between the actual HH values and the HX+HC values. In SHAM rats, the VT responses elicited by HX and HC appeared to be additive since the actual HH values were closely aligned with the computed HX+HC values. In contrast, the VT responses between HX and HC changed markedly in CSNX rats, such that the HX, HC and HH responses were similar to one another, and the actual HH responses were markedly lower than the added HX+HC responses. Therefore, the additive effects of HX and HC signaling pathways were markedly diminished in rats with bilateral CSN transection.

**FIGURE 8:**
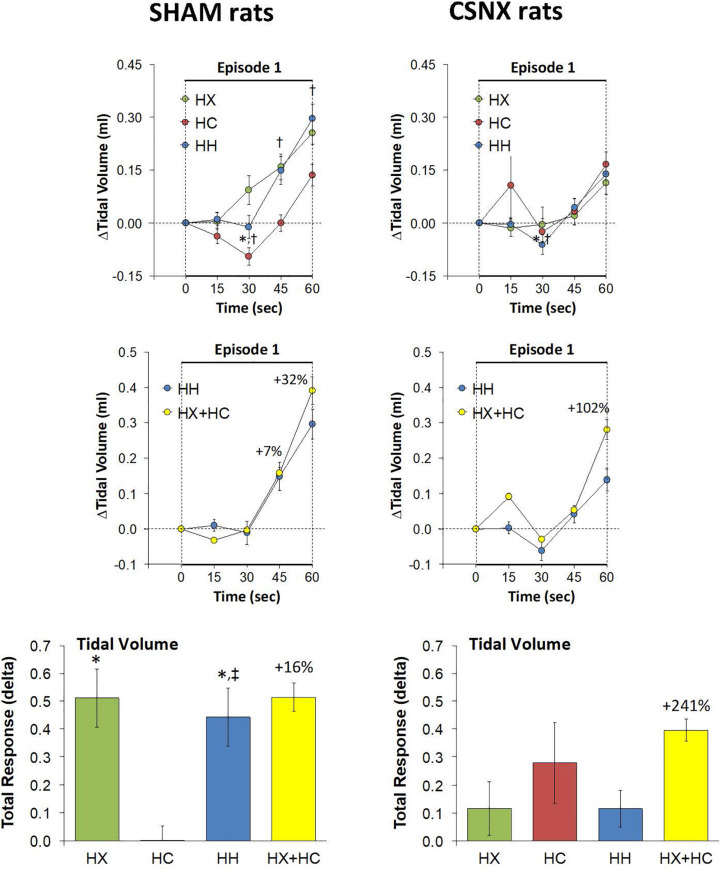
**Top panels:** Arithmetic changes in tidal volume (VT) in freely moving sham-operated (SHAM) rats and in rats with bilateral carotid sinus nerve transection (CSNX) during the first 60 s of exposure to a single hypoxic (HX, 10% O_2_, 90% N_2_), hypercapnic (HC, 5% CO_2_, 21% O_2_, 74% N_2_), or hypoxic-hypercapnic (HH, 5% CO_2_, 10% O_2_, 85% N_2_) gas challenge (Episode 1 (E1)). Data are presented as mean ± SEM. ^∗^*P* < 0.05, HH versus HX. ^†^*P* < 0.05, HH *versus* HC. **Middle panels:** Arithmetic changes in VT in SHAM and CSNX rats during the first 60 s of the actual hypoxic-hypercapnic (HH) gas challenge values compared to addition of the HX and HC (HX+HC) gas challenge values. For the actual HH values, data are shown as mean ± SEM and for HX+HC values, data are presented as the mean ± SEM (10% of mean). **Bottom panels:** Total arithmetic changes in VT in SHAM and CSNX rats during the first 60 s of exposure to HX, HC or HH gas challenges. Data are presented as mean ± SEM. The sum of HX and HC values (HX+HC), expressed as mean ± SEM (10% of mean), are shown. The numbers above the HX+HC columns are the percentage (%) differences between the HX+HC values and actual HH values. ^∗^*P* < 0.05, significant response from Pre-value. ^†^*P* < 0.05, HH *versus* HX. ^‡^*P* < 0.05, HH *versus* HC.

As mentioned, the rapid responses to HX, HC, and HH would be expected to involve CB chemoafferents, and therefore we compared the changes in VT that occurred within the first 60 s of the HX, HC and HH gas challenges in SHAM and CSNX rats. The changes in VT that occurred during the first 60 s of the HX, HC and HH gas challenges are shown in the top panels of Figure 8. For SHAM rats (top left panel), the increases in VT elicited by HC were smaller than those elicited by HX or HH. The magnitude of the initial VT responses elicited by HX, HC and HH in CSNX rats (top right panel) were overall smaller than those in SHAM rats. Unlike the SHAM rats, HX, HC and HH elicited similar initial VT responses in CSNX rats. As seen in the middle panels of Figure 8, the magnitudes of the actual VT responses during the first 60 s of the HH gas challenge were, in general, simply additive of those elicited by HX and HC alone in both SHAM and CSNX rats, except at the 60 s time-point. The bottom panels of Figure 8 summarize the total VT responses that occurred during HX, HC and HH in SHAM (bottom left panel) and CSNX (bottom right panel) rats. Again, for SHAM rats, the initial increases in VT elicited by HH were about that expected by simple addition of HX and HC responses. In contrast, the initial VT responses elicited by HH in CSNX rats were markedly less than expected from simple addition of HX and HC responses. Overall, and in contrast to the fR responses, the VT 60 s data suggests that there is a positive interaction between HX and HC signaling pathways during the first 60 s of a HH gas challenge, and that this interaction is dependent on the presence of the CB-CSN complex.

### Minute Ventilation (MV) in SHAM and CSNX Rats During HX, HC or HH Gas Challenges

**FIGURE 9:**
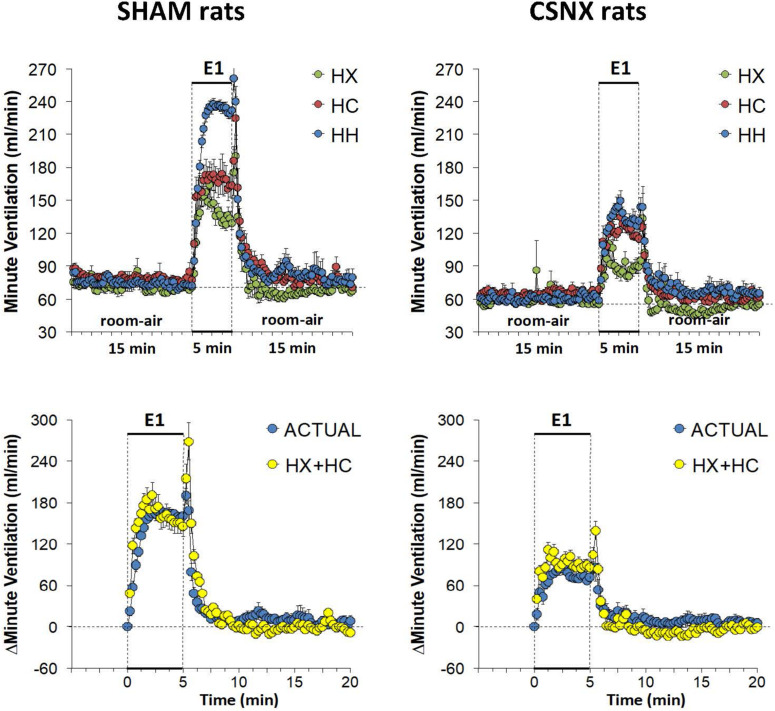
**Top panels:** Minute ventilation (MV) values averaged every 15 s in freely moving sham-operated (SHAM) rats and in rats with bilateral carotid sinus nerve transection (CSNX) during exposure to a single episode (E1) of hypoxic (HX; 10% O_2_, 90% N_2_), hypercapnic (HC; 5% CO_2_, 21% O_2_, 74% N_2_), or hypoxic-hypercapnic (HH; 5% CO_2_, 10% O_2_, 85% N_2_) gas challenge of 5 min in duration, followed by 15 min of room-air. The data are presented as mean ± SEM. **Bottom panels:** Changes in MV in SHAM and CSNX rats during the actual HH gas challenge compared to addition of HX+HC values. For the actual HH values, data are presented as mean ± SEM and for the HX+HC values, data are presented as the mean ± SEM (10% of mean).

The top panels of Figure 9 summarize the MV values recorded in SHAM and CSNX rats before, during, and after exposure to a 5 min HX, HC or HH gas challenge. In SHAM rats (top left panel), the HX and HC challenges elicited robust increases in MV that were relatively similar to one another, except there was a more robust roll-off during HX than HC. The increases in MV during HH were markedly greater than those during HX or HC. HX, HC and HH also elicited reproducible increases in MV in CSNX rats (top right panel) that appeared substantially smaller than those in SHAM rats. In CSNX rats, the increases in MV elicited by HH were similar to those elicited by HC, but greater than those elicited by HX. The bottom panels of Figure 9 summarize the arithmetic changes in MV that occurred during the actual HH gas challenge and from simple addition of HX and HC gas challenge values (HX+HC). The actual HH values and additive HX+HC values were remarkably similar in SHAM rats (bottom left panel) and CSNX rats (bottom right panel). The maximal attainable changes in MV in SHAM rats during the entire HH protocol was +190 ± 6 ml/min (Table 2), which was much higher than the changes during the HH gas challenge (+168 ± 4 ml/min) (Table 2), but similar to the sum of the HX and HC (HX+HC) gas challenge responses (+84 ± 5 ml/min and +99 ± 13 ml/min, respectively; +183 ml/min upon addition). The maximal attainable changes in MV in CSNX rats during the entire HH protocol was +138 ± 6 ml/min (Table 2), which was substantially higher than those observed during the HH gas challenge (+85 ± 6 ml/min), and somewhat higher than the sum of the HX and HC (HX+HC) gas challenge responses (+46 ± 5 ml/min and +70 ± 5 ml/min, respectively; +116 ml/min upon addition).

**FIGURE 10:**
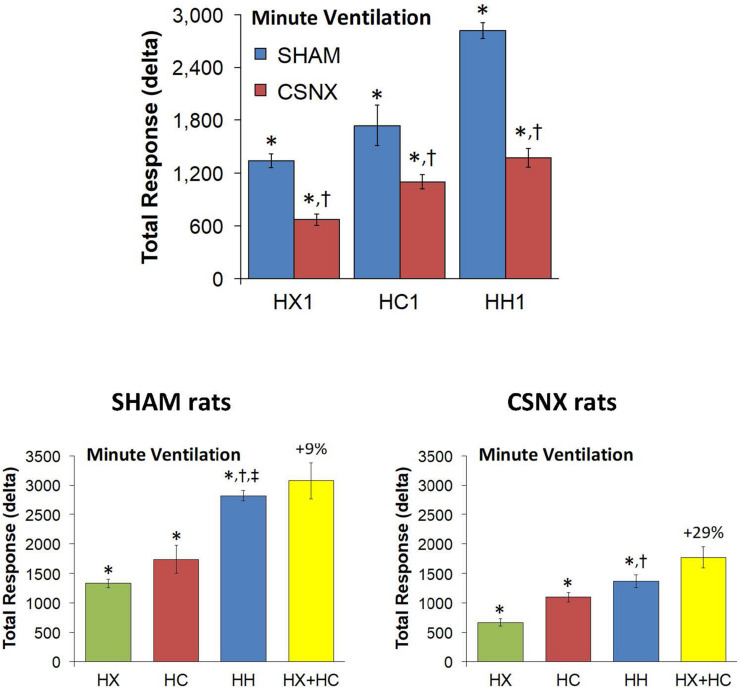
**Top panels:** Total arithmetic changes in minute ventilation (MV) that occurred during exposure to a single hypoxic (HX1, 10% O_2_, 90% N_2_), hypercapnic (HC1, 5% CO_2_, 21% O_2_, 74% N_2_) or hypoxic-hypercapnic (HH1, 5% CO_2_, 10% O_2_, 85% N_2_) gas challenge in SHAM and CSNX rats. The data are presented as mean ± SEM. ^∗^*P* < 0.05, significant response from Pre-value. ^†^*P* < 0.05, CSNX *versus* SHAM. **Bottom panels:** Total arithmetic changes in MV in SHAM (left panel) and CSNX (right panel) rats during exposure to the HX, HC or HH gas challenges. The sum of HX and HC values (HX+HC) expressed as mean ± SEM (10% of mean) are shown. The figures in parentheses above the HX+HC columns are the percentage (%) differences between actual HH values and HX+HC values. ^∗^*P* < 0.05, significant response from Pre-value. ^†^*P* < 0.05, HH *versus* HX. ^‡^*P* < 0.05, HH *versus* HC.

The top panels of Figure 10 compare the total increases in MV that occurred during the single HX, HC or HH gas challenge in SHAM and CSNX rats. The total increases in MV elicited by HX and HC were significantly smaller in CSNX compared to SHAM rats. The total increases in MV elicited by HH were also significantly smaller in CSNX than SHAM rats. Table 3 shows that the arithmetic differences in MV between SHAM rats and CSNX rats for HX of -667 ml/min and for HC of -635 ml/min, adds to -1302 ml/min, and the difference in MV for the actual HH gas challenge was -1448 ml/min, suggesting that the MV difference in HH responses between SHAM and CSNX rats was similar to that predicted by addition of the HX and HC (HX+HC) responses. The bottom panels of Figure 10 summarize the total MV responses elicited by the HX, HC and HH gas challenges in the SHAM rats (bottom left panel) and CSNX rats (bottom right panel). The summed HX+HC values are displayed with figures above the HX+HC columns showing the percentage difference between the actual HH values and HX+HC values. It seems evident that the MV response during HH was simply equal to the sum of the HX and HC (HX+HC) responses in SHAM rats, whereas in CSNX rats, the summed HX+HC responses were slightly greater than the HH response for MV.

**FIGURE 11:**
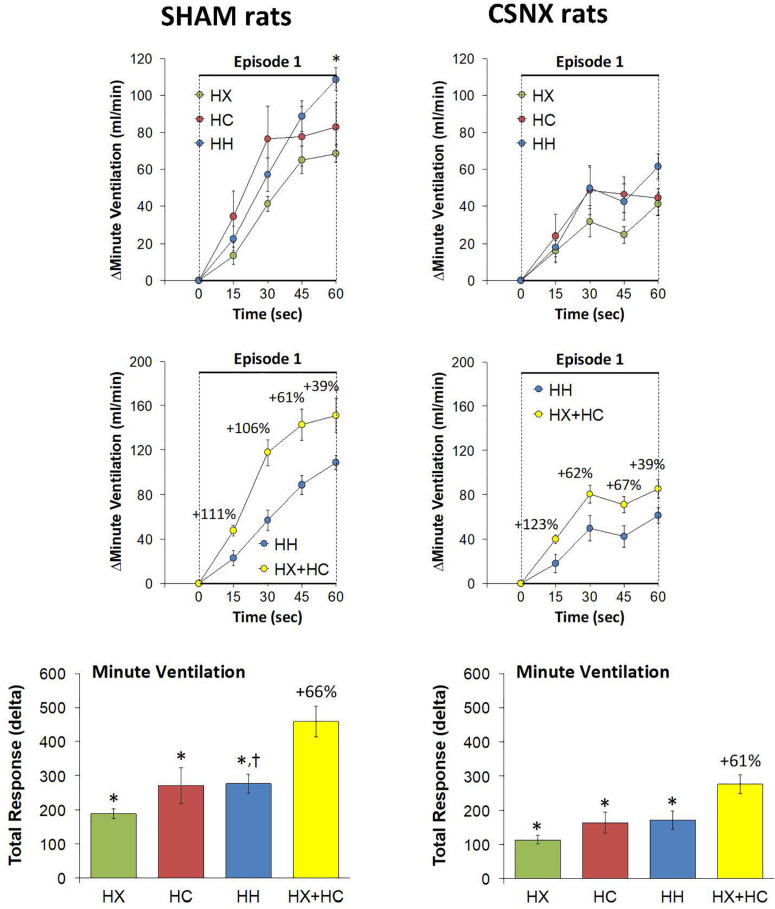
**Top panels:** Arithmetic changes in minute ventilation (MV) in freely moving sham-operated (SHAM) rats and in rats with bilateral carotid sinus nerve transection (CSNX) during the first 60 s of exposure to a single hypoxic (HX, 10% O_2_, 90% N_2_), hypercapnic (HC, 5% CO_2_, 21% O_2_, 74% N_2_), or hypoxic-hypercapnic (HH, 5% CO_2_, 10% O_2_, 85% N_2_) gas challenge (Episode 1 (E1)). Data are presented as mean ± SEM. ^∗^*P* < 0.05, HH versus HX. ^†^*P* < 0.05, HH *versus* HC. **Middle panels:** Arithmetic changes in MV in SHAM and CSNX rats during the first 60 s of the actual hypoxic-hypercapnic (HH) gas challenge values compared to addition of the HX and HC (HX+HC) gas challenge values. For the actual HH values, data are shown as mean ± SEM and for HX+HC values, data are presented as the mean ± SEM (10% of mean). **Bottom panels:** Total arithmetic changes in MV in SHAM and CSNX rats during the first 60 s of exposure to HX, HC or HH gas challenges. The data are presented as mean ± SEM. The sum of the HX and HC values (HX+HC), expressed as mean ± SEM (10% of mean), are also shown. The numbers above the HX+HC columns are the percentage (%) differences between the HX+HC values and actual HH values. ^∗^*P* < 0.05, significant response from Pre-value. ^†^*P* < 0.05, HH *versus* HX.

Again, the initial responses to HX, HC and HH would be expected to be mediated by CB chemoafferents, and so it is important to establish what changes in MV during these challenges occurred within the first 60 s in SHAM and CSNX rats. The changes in MV that occurred during the first 60 s of HX, HC and HH are shown in the top panels of Figure 11. In SHAM rats (top left panel), the initial increases in MV elicited by HX, HC and HH were similar to one another. In CSNX rats (top right panel), the initial increases in MV elicited by HX, HC and HH were also similar to one another, and obviously smaller compared to the SHAM rats. Again, we wanted to determine whether the magnitude of the actual 60 s MV response during HH was simply additive of that elicited by HX and HC alone. As seen in the middle panels of Figure 11, the actual 60 s MV responses during HH in both SHAM and CSNX rats were substantially smaller than those of the computed HX+HC values. The bottom panels of Figure 11, show the total MV responses that occurred during the first 60 s of HX, HC and HH in SHAM rats (bottom left panel) and CSNX rats (bottom right panel). The initial increase in MV elicited by HH was less than would be expected by simple addition of the HX and HC responses in both SHAM and CSNX rats. Thus, as with (and because of) the fR responses, the concluding message of the 60 s data is that there is a negative interaction between HX and HC signaling pathways during the first 60 s of the HH gas challenge, and this is independent of the presence or absence of the CB-CSN complex.

## Discussion

Proper tissue oxygenation is a well-regulated process that is essential for the survival of air-breathing animal species. The CBs are crucial for detecting changes in arterial pO_2_, pCO_2_ and pH. A drop in arterial pO_2_ (hypoxia) or a rise in pCO_2_ (hypercapnia) stimulates the CBs, and the sensory information is then relayed to chemoafferents of the CSN, which in turn sends signals to the respiratory control regions in the brainstem to trigger downstream respiratory and cardiovascular reflexes (López-Barneo et al., 2008). Over the years research has shown developmental differences between hypoxia (HX) and hypercapnia (HC) ventilatory responses in neonatal and adult mammals (Bureau et al., 1984; Bonora et al., 1984; McCooke and Hanson, 1985; Easton et al., 1986; Eden and Hanson, 1987; Vizek et al., 1987; Elnazir et al., 1996; Maxová and Vízek, 2001; Stunden et al., 2001; Putnam et al., 2005; Pokorski and Antosiewicz, 2010; Teppema and Dahan, 2010). In addition, studies have shown that CSNX blunts the HX and HC ventilatory responses in both adult and newborn animals (Chalmers et al., 1967; Sapru and Krieger, 1977; Cardenas and Zapata, 1983; Bureau et al., 1985; Olson et al., 1988; Suguihara et al., 1994; Fung et al., 1996; Lowry et al., 1999a, b; Teppema and Dahan, 2010; Mouradian et al., 2012; Baby et al., 2018). However, the effect of CSNX on HX (poikilocapnic), HC and hypoxia-hypercapnia (HH) ventilatory responses in juvenile (P25) rats remains unclear. Earlier studies have shown that Sprague Dawley rats at P25 are not sexually mature (Sengupta, 2013), but have fully mature peripheral and central neuronal systems (Dumas, 2005; Jiang et al., 2005; Tahayori and Koceja, 2012; Sinclair et al., 2014; Lai et al., 2016). Thus, we used the P25 age group in this study to analyze the role of peripheral CB chemoafferents in the ventilatory performance of juvenile rats. We hypothesized that the peripheral chemosensory pathway in juvenile rats plays a unique role in determining the interaction between O_2_ and CO_2_, and consequently, the response to hypoxic-hypercapnic gas challenges. Our present study provides compelling evidence supporting previous observations that peripheral and central chemoreflexes appear to be fully operational in Sprague Dawley P25 rats, because we found that the HX (10% O_2_, 90% N_2_) and HC (5% CO_2_, 21% O_2_, 90% N_2_) gas challenges we exposed the P25 rats to elicited robust increases in fR, VT and MV in SHAM rats that resemble in many respects those observed in adult rats of various strains, including Sprague Dawley rats (Teppema and Dahan, 2010; May et al., 2013a, b).

HX and HC challenges are probes used to study and determine ventilatory signaling cascades elicited by decreases in arterial pO_2_ or increases in arterial pCO_2_, and the HH challenge is more physiological and clinically relevant in that it causes changes in pO_2_ and pCO_2_ blood-gas chemistry that resemble those elicited by central/obstructive sleep apneas (Smith et al., 1984, 2006, 2010, 2015; Blain et al., 2010; Forster and Smith, 2010; May et al., 2013a, b). The major objectives of this study were (1) to characterize the changes in fR, VT and MV elicited by HX, HC and HH in order to define and compare, how these challenges affect ventilation in juvenile rats; (2) to determine how ventilatory responses elicited by HX, HC and HH are dependent on the presence of CB chemoafferents; (3) to determine the interaction between HX and HC signaling pathways in SHAM rats in order to characterize whether these inputs are (a) simply additive (i.e., independent of one another), (b) synergistic (super-additive) or (c) inhibitory (hypo-additive); and (4) to determine whether the interaction between hypoxia and hypercapnia signaling pathways is under the influence of the CB-CSN chemoafferent complex (i.e., is the interaction disturbed in bilateral CSNX rats).

Our data shows that (1) the initial rate of rise (over the first 60 s) in fR, VT and MV elicited by the HX, HC and HH gas challenges were shallower in CSNX rats than in SHAM rats; (2) the total increases in fR, VT, and MV elicited by HX were diminished in CSNX rats compared to SHAM rats; (3) the total increases in fR, VT and MV elicited by HC in CSNX rats were not significantly different from those in SHAM rats; and (4) the total increases in fR, VT and MV elicited by HH observed in SHAM rats were markedly diminished in CSNX rats. These findings suggests and/or support previous evidence that (1) the activity of CB chemoafferents increases rapidly in response to changes in arterial blood pO_2_ and pCO_2_, thereby allowing more rapid ventilatory responses than central chemoreceptors (Fatemian et al., 2003; Smith et al., 2006), (2) the CB-CSN inputs to the cNTS play a discernible and important role in HX ventilatory signaling, while central chemoreceptors provide most of the steady-state response to elevations in blood pCO_2_ (Prabhakar, 2000; López-Barneo et al., 2008; Teppema and Dahan, 2010; Guyenet and Bayliss, 2015; Guyenet et al., 2019), (3) the loss of CB-CSN complexes does not impair HC-induced enhancement of ventilatory performance, and (4) the VT response that occurs when HX and HC challenges are given simultaneously (i.e., the HH gas challenge), is indeed additive and this additivity is clearly dependent upon the CB-CSN input to the cNTS as well as direct activation of the retrotrapezoid nucleus (RTN) (Smith et al., 1984, 2006, 2010, 2015; Blain et al., 2010; Forster and Smith, 2010). However, it must be noted that the interactions of the cNTS and RTN with other key nuclei within the brainstem will most likely be of vital importance to the expression of the observed ventilatory responses. Moreover, the relative role of astrocytes and neurons in the brainstem with respect to hypercapnic signaling and the interaction of hypercapnic and hypoxic signaling pathways must also be considered (Sheikhbahaei et al., 2016; Turovsky et al., 2016).

Furthermore, in SHAM rats, the total responses that occurred during the actual HH gas challenge were equal to simple addition of actual HX and HC values (i.e., HX+HC responses). The exception was that the initial increases in fR elicited by HH were less than would be predicted by addition of the HX and HC responses. Thus, our evidence for additivity is consistent with a previous study done in cats showing that when HX and HC stimuli are given together (i.e., HH stimuli), the interaction between central and peripheral chemoreceptors is additive in that one stimulus, HX or HC, simply adds to the other (Eldridge et al., 1981). However, our observation for additivity in P25 rats contradicts previous findings that (1) a positive (synergistic) interaction between hypoxia- and hypercapnia-induced neuronal signaling in cats exists at the level of the CB (Eyzaguirre and Lewin, 1961); (2) in adult dogs (no relevant data in adult rats) that peripheral CB and central chemoreceptors are functionally inter-dependent such that activity and/or sensitivity of medullary chemoreceptors is determined by input from CB chemoreceptors and *vice versa* (Fencl et al., 1966; Smith et al., 1984, 2006, 2010, 2015; Dempsey, 2005; Blain et al., 2010; Forster and Smith, 2010); and (3) interaction between central and peripheral chemoreceptors in cats and rabbits is negative (i.e., the stimuli interact in a hypo-additive manner) (Eldridge, 1974; Kiwull-Schöne et al., 1976). In addition, it is evident from this study that fR, VT and MV responses elicited by HH did not reach the maximum values (i.e., ceiling values) that the rats were capable of achieving throughout the entire 5 × 5 HX, HC or HH gas challenge (i.e., maximum values observed when the rats are grooming, exploring, or in a possible state of elevated vigilance in response to an unexpected external stimulus, such as a sound that the rats could hear, remembering that they were housed in plexi-glass chambers in a quiet room to control for external noise disturbances). Indeed, the markedly smaller than expected increase in fR elicited by HH gas challenge was not simply because the rats could not attain higher values. These findings therefore suggest that HH only recruits ventilatory pathways that have a set-point with respect to the maximum levels of responses that can be achieved.

It should be noted that the P25 rats used for these plethysmography studies were awake and quietly resting before and during the HX, HC and HH gas challenges. This is important to mention because the awake-sleep state of the rat can influence the magnitude of ventilatory responses to direct brain stimulation and in response to HX and HC gas challenges (Abbott et al., 2013). In particular, the ventilatory responses seen during awake and non-REM sleep stages are similar, but markedly blunted during REM sleep. In our studies, none of the rats were asleep at any stages of the study and as such we do not believe that the data was influenced by the awake-sleep status of the rats. In addition, it should be noted that robust ventilatory responses to a poikilocapnic HX gas challenge were evident in the juvenile (P25) rats with prior bilateral CSNX. This finding differs strikingly to the much more dramatic loss of ventilatory responses to HX gas challenge in adult rats and mice (Bisgard et al., 1976; Sapru and Krieger, 1977; Smith and Mills, 1980; Martin-Body et al., 1985, 1986; Olson et al., 1988; Roux et al., 2000; Baby et al., 2018). Whether the remaining ventilatory responses to HX challenge in P25 rats with bilateral CSNX is due to (1) low secondary O_2_-sensing structures, such as aortic bodies or peripheral (e.g., glossopharyngeal) nerves (Eyzaguirre and Lewin, 1961); (2) the dynamic functional reorganization of central chemoreceptor reflex pathways (Roux et al., 2000); and/or (3) central O_2_ sensors (Dillon and Waldrop, 1993; Sun and Reis, 1994), remains to be determined. Finally, our data suggesting that loss of CSN input to the brainstem markedly impairs the additivity of the hypoxic-hypercapnic ventilatory response, may have important implications for the development of disease processes whereby the loss of function of the CBs and/or CSN chemoafferents contributes to changes in ventilation in unexpected ways.

### Study Limitations

An important limitation of this study is that it only provides a snapshot of the temporal changes in ventilatory responses to HX, HC and HH gas challenges after bilateral CSNX. The 4-day recovery period was chosen because we wanted to expose the P25 rats to HX, HC and HH challenges before the diminished ventilatory responses to HX challenges are known to return to their full expression, which is about 2-3 weeks post-CSNX (Bisgard et al., 1976; Smith and Mills, 1980; Martin-Body et al., 1985, 1986; Sinclair, 1987; Dillon and Waldrop, 1993). Another limitation is that although we used standard HX (10% O_2_, 90% N_2_), HC (5% CO_2_, 21% O_2_, 74% N_2_) and HH (10% O_2_, 5% CO_2_, 85% N_2_) gas mixtures, important information may have been obtained with different combinations of these gases. In addition, the lack of female rats in this study is noted and will be rectified in future studies to determine whether female rats of age P25 show different responses to males at a time when sex hormones are not major drivers of physiological status. Finally, we need to mention that baroreceptor afferents within the CSN combine with those in the aortic depressor nerve to regulate arterial blood pressure (Del Rio et al., 2016), and therefore another important limitation of the present study is that we did not assess the cardiovascular responses, sympatho-vagal balance or baroreceptor reflex activity before or during the gas challenges in the SHAM or CSNX rats. The role of the baroreceptor afferents within the CSN and the consequences of their loss following bilateral CSNX to ventilatory responses of HX, HC and HH gas challenges will be looked at in future studies.

## Conclusion

Overall, this novel data reveals that the CSN provides necessary input to respiratory control regions in the brainstem of juvenile (P25) rats in order to induce HX, HC and HH ventilatory responses. In addition, our data suggests that juvenile rats with prior CSNX possess a functional secondary means of low O_2_ sensing, other than the CB-CSN complex, that also send signals to respiratory control centers of the brainstem to trigger downstream respiratory reflexes. Finally, our results reveal that additive between HX and HC stimuli is absent in rats with CSNX, therefore suggesting that loss of CSN chemoafferent signaling to the brainstem allows central CO_2_ chemoreceptors to dominate the low secondary O_2_ sensing input at the points of convergence between the central and peripheral chemoreceptor input.

**FIGURE 12:**
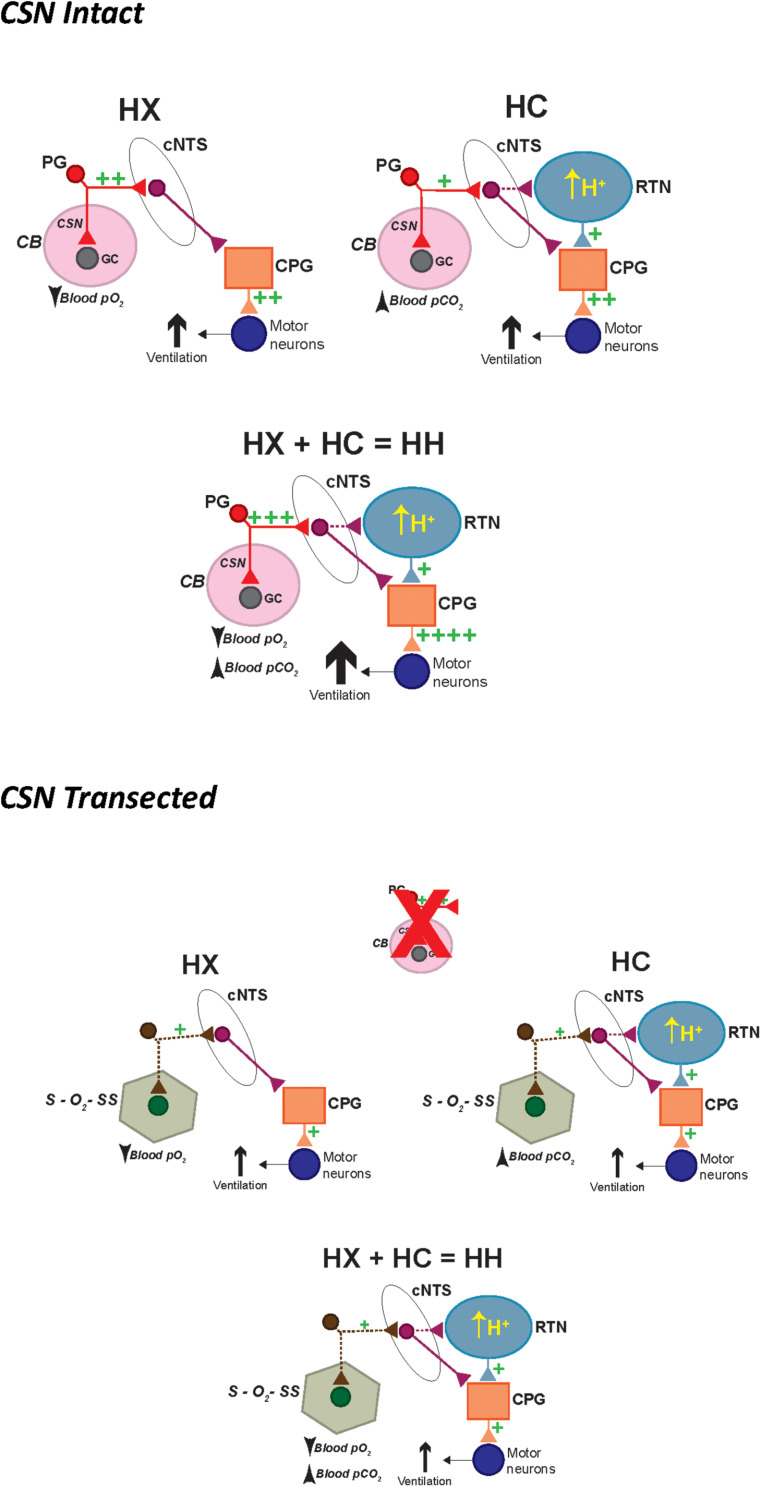
Schematic describing how activation of hypoxic and hypercapnic signaling pathways increase ventilation in P25 male SD rats in a simple additive manner and how this additivity is lost with prior CSN transection. HX, hypoxic gas challenge. HC, hypercapnic gas challenge. HH, hypoxic-hypercapnic gas challenge. CB, carotid body. GC, glomus cell. CSN, carotid sinus chemoafferent nerve. PG, petrosal ganglion cell body of the chemoafferent nerve. cNTS, commissural nucleus tractus solitarius. RTN, retrotrapezoid nucleus. CPG, central pattern generator.

In conclusion we have comprised a series of schematics describing how HX and HC signaling pathways increase ventilation in P25 male rats with the CSN intact and CSN transected (Figure 12). In SHAM rats (i.e., CSN intact), a hypoxic gas challenge (HX) triggers a *strong* (++) depolarization of chemosensitive glomus cells (GC) in the CB that causes the release of excitatory neurotransmitters. These neurotransmitters then activate chemosensory afferent fibers in the CSN, whose cell bodies are located in the petrosal ganglion (PG). The central projections of these chemoafferents signal neurons in the commissural nucleus tractus solitarius (cNTS), which relay downstream respiratory-related neuronal pathways in the central pattern generator (CPG) to increase ventilation (++) in an effort to restore blood gas homeostasis. A hypercapnic gas challenge (HC) triggers a *moderate* (+) depolarization, and release of neurotransmitters from chemosensitive GC in the CB. In addition, HC triggers a *moderate* (+) depolarization and release of neurotransmitters from central chemosensors in the retrotrapezoid nucleus (RTN). Both signaling pathways converge in the CPG to ultimately increase ventilation (++). A hypoxic-hypercapnic gas challenge (HH) triggers a *larger* (+++) depolarization compared to HX and release of neurotransmitters from chemosensitive GC in the CB. This is due to the positive interaction between hypoxia and hypercapnia when given together at the level of the CB. In addition, HH triggers a *moderate* (+) depolarization and release of neurotransmitters from central chemosensors in the RTN. Both signaling pathways converge in the central pattern generator (CPG) to ultimately cause an even greater increase ventilation (++++) that is additive of the HX and HC signaling pathways.

In CSNX rats (i.e., CSN transected), HX triggers secondary O_2_-sensing structures (S–O_2_–SS) that, for the sake of argument, may synapse within the cNTS. This S–O_2_–SS input causes a *moderate* (+) activation of cNTS neurons compared to the *strong* (++) activation of the CSN chemoafferent input. The cNTS then signals downstream respiratory-related neuronal pathways in the CPG to increase ventilation (+). A HC triggers a *smaller* (+) depolarization, and release of neurotransmitters from S–O_2_–SS, compared to CSN chemoafferent input. In addition, it triggers a *moderate* (+) depolarization from central chemosensors in the RTN. Both pathways converge in the CPG with the input from the RTN occluding the input from the S–O_2_–SS. As such, the increase in ventilation (+) is mediated solely by the central chemoreceptors, and thus the additivity that is seen when the CSN is intact is lost. Finally, HH triggers a *smaller* (+) depolarization, and release of neurotransmitters from S–O_2_–SS, compared to CSN chemoafferent input. In addition, it triggers a *moderate* (+) depolarization from central chemosensors in the RTN. Both pathways converge in the CPG with the input from the RTN occluding the input from the S–O_2_–SS. As such, the increase in ventilation (+) is mediated solely by the central chemoreceptors, and thus the additivity that is seen when the CSN is intact is again lost. We understand that these schematics are rudimentary, and that there are many other plausible possibilities to explain our data for (1) the additivity of HX and HC signaling pathways in SHAM rats; and (2) the loss of this additivity in CSNX rats. We are sure that this series of schematics will evolve as new data is collected.

## Data Availability Statement

The raw data supporting the conclusions of this article will be made available by the authors, without undue reservation.

## Ethics Statement

The animal study was reviewed and approved by the Case Western Reserve University Animal Care and Use Committee.

## Author Contributions

PG and SL conceived and designed the study, analyzed the data, and prepared the figures. PG and GC performed the surgeries and plethysmography studies. All authors contributed to writing the manuscript, and revised, read, and approved the final version of the manuscript.

## Conflict of Interest

The authors declare that the research was conducted in the absence of any commercial or financial relationships that could be construed as a potential conflict of interest.

## Abbreviations

CB: carotid body
CSN: carotid sinus nerve
CSNX: carotid sinus nerve transection
Freq: frequency of breathing
HC: hypercapnia
HH: hypoxia-hypercapnia
HX: hypoxia
MV: minute ventilation
NTS: nucleus tractus solitarius
RTN: retrotrapezoid nucleus
VT: tidal volume.
